# Der erste Modellstudiengang Zahnmedizin in Deutschland – iMED DENT

**DOI:** 10.1007/s00103-023-03801-5

**Published:** 2023-11-03

**Authors:** Andreas H. Guse, Thomas Beikler, Guido Heydecke, Martin Gosau, Bärbel Kahl-Nieke

**Affiliations:** 1 Dekanat der Medizinischen Fakultät, Universitätsklinikum Hamburg-Eppendorf, Martinistr. 52, 20246 Hamburg, Deutschland; 2 Institut für Biochemie und Molekulare Zellbiologie, Universitätsklinikum Hamburg-Eppendorf, Hamburg, Deutschland; 3 Poliklinik für Parodontologie, Präventive Zahnmedizin und Zahnerhaltung, Universitätsklinikum Hamburg-Eppendorf, Hamburg, Deutschland; 4 Poliklinik für Zahnärztliche Prothetik, Universitätsklinikum Hamburg-Eppendorf, Hamburg, Deutschland; 5 Klinik für Mund‑, Kiefer- und Gesichtschirurgie, Universitätsklinikum Hamburg-Eppendorf, Hamburg, Deutschland; 6 Poliklinik für Kieferorthopädie, Universitätsklinikum Hamburg-Eppendorf, Hamburg, Deutschland

**Keywords:** Zahnmedizin, Modellstudiengang, Studiengangziele, Modulare Studiengangstruktur, Qualitätssicherung, Dentistry, Model study program, Study program objectives, Modular study program structure, Quality assurance

## Abstract

Im Oktober 2019 startete mit iMED DENT der erste Modellstudiengang Zahnmedizin in Deutschland. Dem Start ging ein mehrjähriger Entwicklungsprozess voraus, in dem anfangs u. a. europäische Standorte mit innovativen Zahnmedizinstudiengängen besucht wurden. Danach wurde das zentrale Reformziel des Modellstudiengangs festgelegt: die Entwicklung, Implementierung und fortlaufende Optimierung eines interdisziplinären Curriculums mit Wissenschaftsbezug, das theoretische und praktische zahnmedizinische Inhalte integriert. Weitere Schritte waren die Entwicklung der Studiengangziele sowie der modularen Studienstruktur. Letztere besteht aus den 3 Teilen „Normalfunktion“, „Vom Symptom zur Erkrankung“ und „Therapie“. Im Curriculum wird der zentrale Bereich der Zahnmedizin von den medizinischen Grundlagenfächern und den klinisch-medizinischen Fächern flankiert. Der vorliegende Beitrag berichtet über die wichtigen Entwicklungsschritte des Modellstudiengangs, seine Struktur und qualitätssichernde Maßnahmen. Erste Auswertungen zur Erreichung von Studiengangzielen bzw. zum Optimierungsbedarf im laufenden Curriculum werden vorgestellt.

## Abstract (en)

October 2019 saw the launch of iMED DENT, the first model study program in dentistry in Germany. The launch was preceded by a development process lasting several years in which European locations, among others, with innovative dental study programs were initially visited. The central reform objective of the model study program was then defined: the development, implementation, and ongoing optimization of an interdisciplinary curriculum with a scientific focus that integrates theoretical and practical dental content. Further steps were the development of the study program objectives and the modular study structure. The latter consists of the three parts: “Normal Function,” “From Symptom to Disease,” and “Therapy.” In the curriculum, the central area of dentistry is flanked by basic and clinical medical subjects. This article reports on the important development steps of the model study program, its structure, and quality assurance measures. First evaluations of the achievement of study program objectives and the need for optimization in the current curriculum are presented.

## Einleitung

„Wie steht es um die Mundgesundheit in Deutschland?“ Diese Frage wurde 2021 in den Ausgaben 7 und 8 des Bundesgesundheitsblattes gestellt [1, 2]. Inzwischen wurde sie durch die Resolution zur Mundgesundheit des Exekutivrats der Weltgesundheitsorganisation (WHO) 2021 [3] ebenso wie durch das Strategiepapier „FDI Vision 2030“ des Weltzahnärzteverbandes (Fédération Dentaire International – FDI; [4]) beantwortet. Beide Institutionen betonen die Notwendigkeit, die Mundgesundheit als essenziellen Bestandteil der universellen Gesundheitsversorgung zu stärken.

Um die Versorgung der Mundgesundheit der Bevölkerung auf hohem Niveau zu gewährleisten, stellen die fortwährende Weiterentwicklung von Aus‑, Fort- und Weiterbildung in der Zahnmedizin sowie der nachhaltige Ausbau geeigneter Strukturen für zahnmedizinische Versorgungsforschung an den Universitäten essenzielle Bausteine zur Verbesserung der Mundgesundheit dar [1].

Der Modellstudiengang Zahnmedizin iMED DENT fußt auf dem Zahnheilkundegesetz (ZHG) in seiner bis 2019 gültigen Version. Der damals noch enthaltene § 3a ermöglichte „Zur Anpassung des Studiums der Zahnmedizin an die fachliche Weiterentwicklung der Zahnmedizin … bei der nach Landesrecht zuständigen Stelle die Zulassung eines Modellstudiengangs [zu] beantragen“ (Satz 1). Die Sätze 2 und 3 des § 3a regelten mögliche Abweichungen von den Vorgaben der Approbationsordnung für Zahnärzt:innen (Satz 2) sowie die Zulassungsvoraussetzungen für einen Modellstudiengang (Satz 3). Die wichtigste inhaltliche Voraussetzung für die Zulassung eines Modellstudiengangs Zahnmedizin war, dass „das Reformziel beschrieben wird und erkennen lässt, welche qualitativen Verbesserungen für die zahnmedizinische Ausbildung vom Modellstudiengang erwartet werden“.

Das zentrale Reformziel des Modellstudiengangs iMED DENT bestand und besteht in der *Entwicklung, Implementierung und fortlaufenden Optimierung eines theoretische und zahnmedizinisch praktische Inhalte integrierenden, interdisziplinären Curriculums mit Wissenschaftsbezug*. Unterstützende Rahmenbedingungen zur Erreichung dieses Reformziels waren (i) die bereits erfolgte Einführung und Optimierung der integrierten klinischen Kurse (IK) im 4. und 5. Studienjahr in der regelhaften Ausbildung der Studierenden der Zahnmedizin in Hamburg sowie (ii) die durchweg ermutigenden Erfahrungen seit Einführung des Modellstudiengangs Medizin iMED mit vergleichbarem curricularen Konzept.

Der nachfolgende Bericht beschreibt wichtige Entwicklungsschritte des Curriculums, die dann aus dem zentralen Reformziel (s. oben) abgeleiteten Studiengangziele, die Struktur des Studiengangs, eine Auswahl qualitätssichernder Maßnahmen sowie erste Daten zur Erreichung von Studiengangzielen bzw. zum Optimierungsbedarf im laufenden Curriculum.

## Entwicklung des Curriculums

Nachdem die medizinische Fakultät der Universität Hamburg (UHH) bereits im Jahr 2012 den integrierten Modellstudiengang iMED entwickelt und umgesetzt hatte, fanden ab 2016 die ersten Vorbereitungen für den Modellstudiengang iMED DENT statt. Der Bedarf für ein modernes Zahnmedizinstudium, dass dem zahnärztlichen Alltag gerecht wird, kann am besten mit dem Titel „Zahnärztliche Ausbildung zur Förderung und Erhaltung der Mundgesundheit über den gesamten Lebensweg“ beschrieben werden.

Die operative Umsetzung des Reformprojekts begann mit der Einsetzung der Projektsteuergruppe iMED DENT durch den Dekan und den Prodekan für Lehre. Sie umfasste die Fachvertreter:innen aus Zahnmedizin und vorklinischer, theoretisch-klinischer und klinischer Medizin. Medizindidaktisch fortgebildete Lehrende, z. B. mit dem Studienabschluss „Master of Medical Education“ (MME), unterstützten die Reformbemühungen mit innovativen didaktischen Konzepten. In regelmäßigen Treffen wurde das weitere Vorgehen festgelegt; hier sei auch die sehr wichtige Funktion von häufigen, mehrtägigen „Retreats“ außerhalb des Universitätsklinikums genannt, bei denen die z. T. unterschiedlichen Vorstellungen zur curricularen Entwicklung in Kompromisslösungen überführt und konsentiert wurden.

Zunächst fanden auf Basis einer umfangreichen Literaturrecherche im europäischen Umfeld [5] Vor-Ort-Besuche an 4 Standorten statt. Die Auswahl fiel auf die Standorte Universität Bern, King’s College London, Malmö University und Radboud University Nijmegen. Hier konnte das o. g. Reformziel in seiner Umsetzung vor Ort erlebt und diskutiert werden.

Beispielhaft sei hier aus der Universität Malmö berichtet, die bereits seit vielen Jahren einen auf den Prinzipien des problemorientierten Lernens (POL) basierenden zahnmedizinischen Studiengang betreibt [6]. Beeindruckend war für die Hamburger Besuchsgruppe die Selbstverständlichkeit, mit der Studierende bereits in den ersten Semestern ihr eigenes Arbeitsprogramm auswählen und strukturieren. Auch die Einbindung der Studierenden in den Studienprozess durch Übernahme von Verantwortlichkeit für Untergruppen im Sinne von Koordination, Bewältigung der (selbst-)gestellten Studienaufgaben und der rechtzeitigen Fertigstellung der Lernergebnisse verdeutlichte den hohen Innovationsgrad des Studiengangs in Malmö.

Eine zweite, ausgesprochen relevante, aus dem King’s College mitgebrachte Erfahrung ist die Selbstbewertung von Studierenden aus Vorklinik und Klinik nach einer Methode, über die bereits 2017 publiziert wurde [7]. Sie basiert auf den schon 2011 beschriebenen positiven Effekten für die Lernkultur ([8]; siehe auch Beitrag von Heydecke und Mirzakhanian in diesem Themenheft).

Die Eindrücke aus den Besuchen bei den innovativen europäischen Zahnmedizinstudiengängen wurden von der Projektsteuerungsgruppe iMED DENT aufgenommen und im Zusammenhang mit den bereits vorhandenen Reformideen (z. B. früher Kontakt zu Patient:innen, Wissenschaftsbezug des Curriculums) diskutiert. Die Reformideen mussten dabei mit den gesetzlichen Vorgaben in Deutschland (damaliger § 3a des ZHG, siehe oben) abgeglichen werden, was sich trotz der in Satz 2 gewährten Abweichungsmöglichkeiten von den Vorgaben der Approbationsordnung für Zahnärzte und Zahnärztinnen (ZApprO) als schwierig erwies, da nur wenig Raum für Innovation gelassen wurde. Die Projektsteuergruppe definierte dann die Studiengangziele und entwickelte die curriculare Struktur (siehe unten).

Während der weiteren Entwicklung von iMED DENT wurden alle im Nationalen Kompetenzbasierten Lernzielkatalog Zahnmedizin (NKLZ) gelisteten Lernziele bezüglich ihrer Relevanz für iMED DENT in einem kritischen Diskussionsprozess innerhalb der Fakultät ausgewählt. Dabei standen die für die Vertreter:innen der zahnmedizinischen Fächer fachlich wichtigen Lernziele im Vordergrund. Auch die Vertreter:innen klinisch-medizinischer Fächer und der Grundlagenfächer wurden konsultiert. Aus diesem Abstimmungsprozess mit der Projektsteuergruppe sind dann die Lernziele von iMED DENT hervorgegangen. Sie bilden die inhaltliche Grundlage des Curriculums.

Parallel wurde das Projekt mit den zuständigen Behörden der Freien und Hansestadt Hamburg abgestimmt und dort im Wesentlichen positiv aufgenommen.

## Studiengangziele

### Integration, Interdisziplinarität und Interprofessionalität.

Zentrales Leitbild des Studiums sind die themen- und symptombezogene Vernetzung der zahnmedizinischen Disziplinen untereinander sowie die themen- und symptombezogene Vernetzung der zahnmedizinischen Disziplinen mit den medizinischen Grundlagenfächern und den klinischen Fächern der Medizin, u. a. durch frühe klinische Bezüge mit geeigneten Patientenbeispielen.

### Problem- und symptombezogenes Lernen und Lehren.

Durch Umschichtung von Lehrveranstaltungen soll zur Integration problemorientierter und symptombezogener Unterrichtsformen Raum geschaffen werden, vor allem durch erweitertes problemorientiertes Lernen (POL) im Rahmen eines synoptischen Behandlungskonzepts (alle relevanten Disziplinen inkludierend).

### Früher Patient:innenkontakt.

Durch die Etablierung bzw. Intensivierung „interstudentischer“ klinischer Übungen sollen frühe klinische Erfahrungen ermöglicht werden (z. B. üben Studierende die Untersuchung des Mundraums unter Aufsicht aneinander oder nehmen gegenseitig Abdrücke für die Herstellung von Gipsmodellen). Durch Assistenzprogramme soll eine vertikale Vernetzung der semesterweisen Behandlungskurse stattfinden.

### Wissenschaftliche Orientierung.

Ziel des Studienganges ist auch die Ausbildung von wissenschaftlichen Kompetenzen, d. h. im Wesentlichen, dass die Absolvent:innen

- als lebenslang Lernende professionelles Handeln und stetiges Weiterlernen aufrechterhalten und verbessern,
- Prinzipien und Methoden der evidenzbasierten Medizin und Zahnmedizin anwenden,
- die Evaluation wissenschaftlicher Informationen und Quellen erlernen,
- die Grundsätze guter wissenschaftlicher Praxis befolgen,
- als Lehrende und Multiplikatoren fungieren und
- einen eigenständig verfassten, wissenschaftlichen Beitrag leisten.

In diesem Zusammenhang sind ein weiteres Ziel des Studienganges die Auswahl und Förderung wissenschaftlich besonders geeigneter Studierender.

### Kommunikation.

Im Studiengang iMED DENT werden theoretisches Wissen und praktische Fertigkeiten im Bereich Kommunikation sowie deren zentrale Bedeutung für das spätere ärztliche Handeln vermittelt. Dazu kommen moderne Lehrformate (z. B. Schauspielpatient:innen) zum Einsatz. Das „Kommunikationstraining“ ist als longitudinaler Strang mit zunehmendem Komplexitätsgrad abgebildet. Inhalte des longitudinalen Strangs umfassen Lehr- und Lerninhalte sowohl zur Arzt-Patienten-Kommunikation als auch zu kollegialer und interprofessioneller Kommunikation und sollen somit zu einem respektvollen Umgang unter Kommiliton:innen, zwischen Lehrenden und Studierenden sowie zwischen Kolleg:innen und zu einer professionellen Feedbackkultur führen.

## Studienstruktur

### Grundlagen- und Nebenfächer flankieren den Kernbereich Zahnmedizin

**Figure 1:**
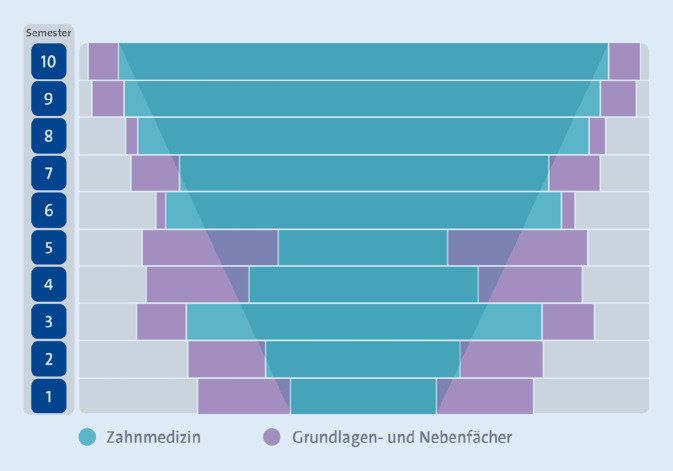

Wesentliches Strukturmerkmal des Curriculums ist die über den gesamten Studienverlauf eng verzahnte theoretische und klinisch-praktische Ausbildung. Der v‑förmige Aufbau von iMED DENT (Abb. 1) ergibt sich dadurch, dass der Kernbereich Zahnmedizin von den Grundlagen- und Nebenfächern flankiert wird, die zu Beginn des Studiums noch mehr Raum einnehmen als zum Ende hin, während die Anteile der zahnmedizinischen Fächer im Verlauf zunehmen. Theoretische, theoretisch-klinische und klinisch-praktische Fächer aus der Medizin sind immer dann in integrierten Lehrformaten präsent, wenn dies inhaltlich erforderlich und für das Verständnis der zahnmedizinischen Inhalte unabdingbar oder förderlich ist. In allen Modulen des Studiums werden theoretische Grundlagen, praktische Kompetenzen in Diagnose und Therapie sowie soziale Interaktion mit Patient:innen und Kolleg:innen parallel vermittelt (siehe nächster Abschnitt).

**Figure 2:**
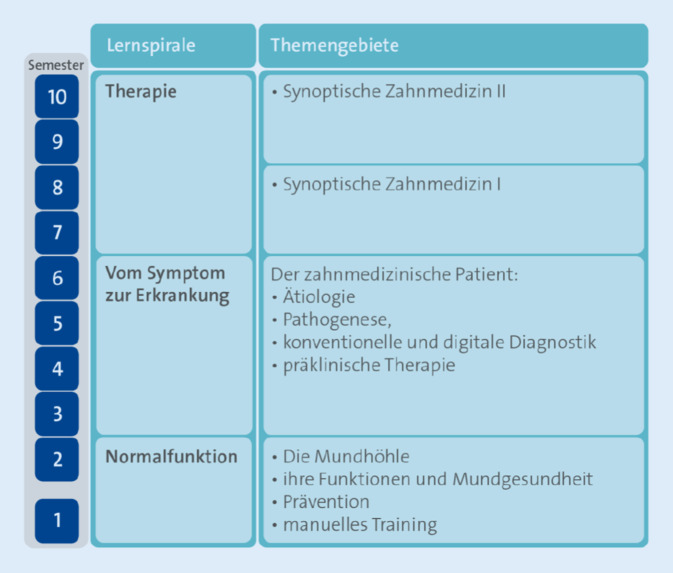

Im Kernbereich „Zahnmedizin“ sieht das Curriculum als Grundprinzip eine wissenschaftsbasierte und an zahnärztlichen und psychosozialen Kompetenzen orientierte Lernspirale vor, die Theorie und Klinik integriert (Abb. 2). Diese sich in ihren Anforderungen steigernde Lernspirale reicht vom *wissenschaftlichen Verständnis der gesunden Strukturen und Funktionen* von Zähnen, Kiefer, Mundhöhle und angrenzenden Bereichen sowie des Körpers im Allgemeinen über das *evidenzbasierte Verständnis von Krankheit* bis hin zum *zahnärztlich-diagnostischen, -therapeutischen und betreuenden Handeln*. Sie beinhaltet eine fundierte Ausbildung zum wissenschaftlichen Arbeiten, die mit einer Studienarbeit abgeschlossen wird, sowie die Möglichkeit einer sich anschließenden studienbegleitenden Promotion zum Dr. med. dent. Die approbierten Zahnärzt:innen werden dadurch zur lebenslangen Weiterbildung befähigt. Die Lernspirale berücksichtigt ein interdisziplinäres Krankheitsverständnis sowie longitudinale Aspekte (z. B. Lebensphasen, psychosoziale Medizin und Sozialmedizin, Schmerz- und Palliativmedizin, Gender-Aspekte). Und sie ermöglicht Auslandsaufenthalte im Bereich der Krankenversorgung und/oder der Forschung. Insgesamt sind 3 Studienabschnitte zu absolvieren. Dem 1. Studienabschnitt *„Normalfunktion“* im 1. Studienjahr folgen die Abschnitte 2 *„Vom Symptom zur Erkrankung“* (2. und 3. Jahr) und 3 „*Therapie*“ (4. und 5. Jahr; Abb. 2).

Im 1. Studienabschnitt erfolgt die Grundausbildung „Normalfunktion“, die sich im Wesentlichen am zahnmedizinisch gesunden Menschen orientiert und zusätzlich bereits erste Aspekte der zahnklinisch-praktischen Ausbildung beinhaltet. Im Mittelpunkt stehen dabei der Aufbau und die Funktionen der Mundhöhle, die Mundgesundheit und präventive Maßnahmen zu deren Erhaltung sowie erste manuelle Trainingseinheiten.

Der Übergang zum patientenzentrierten Unterricht findet im 2. Studienabschnitt statt, mit der Ätiologie und Pathogenese zahnmedizinischer Erkrankungen sowie den diagnostischen und therapeutischen Optionen. Die in den Unterricht integrierte Ausbildung erfolgt hier am Modell (Phantom) bzw. durch „interstudentische“ klinische Übungen. Zahnmedizinische Befunde werden genutzt, um den klinisch-praktischen Anteil auszubauen.

Im 3. Studienabschnitt steht dann die synoptische Zahnmedizin im Zentrum, in der sowohl die theoretischen als auch alle praktischen Anteile der Zahnmedizin an Patient:innen erlernt werden. Vermittelt wird ein an klinischen Kompetenzen orientiertes Verständnis der zahnmedizinischen Therapie. Dieser Teil wird in den zahnmedizinischen Fächern Zahnerhaltung, Prothetik, Kieferorthopädie sowie Mund‑, Kiefer- und Gesichtschirurgie angeboten (siehe auch Beiträge von Bender et al., Lemke et al. und Mirzakhanian und Heydecke in diesem Themenheft).

### Modularisierung der Lehrveranstaltungen

**Figure 3:**
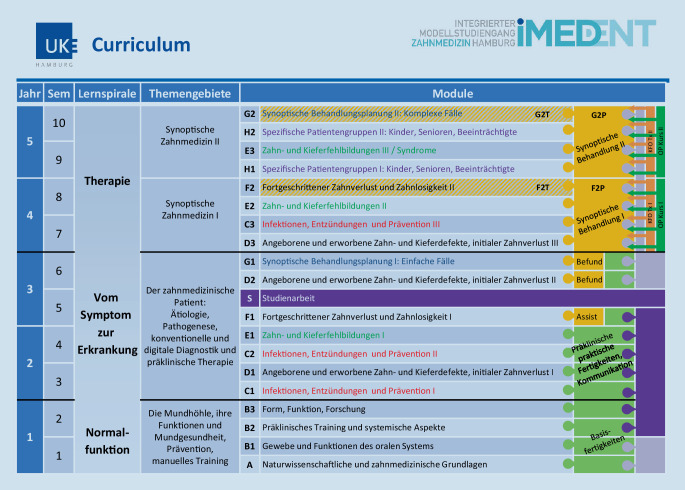

Das zweite wesentliche Strukturmerkmal von iMED DENT ist die Modularisierung der Lehrveranstaltungen (Abb. 3). Dabei sind 2–3 Module jeweils thematisch zu Modulblöcken zusammengefasst. Diese Zusammenfassung erlaubt, ähnlich wie im Modellstudiengang Medizin iMED [9, 10], übergreifende Themen wiederholt im Unterricht darzustellen und damit einen nachhaltigeren Lernprozess zu fördern. Pro Semester sind 2 je 7‑wöchige Module vorgesehen. Die Modulabschlussprüfungen sind in die jeweiligen Module integriert. Die besondere Struktur der „Module“ F2P und G2P wird bei der Beschreibung der einzelnen Modulblöcke im Folgenden erläutert. Die Themen der Modulblöcke A bis H und S werden im Folgenden beschrieben.

#### Modulblock *A: Naturwissenschaftliche und zahnmedizinische Grundlagen.*

Die im singulären Modulblock A vermittelten und erlernten Lehrinhalte sind unverzichtbare naturwissenschaftliche und zahnmedizinische Grundlagen, deren Kenntnis Voraussetzung für alle folgenden Modulblöcke ist, sodass sie dort wiederholt und vertieft werden sowie als Anwendungsgrundlage fungieren.

#### Modulblock *B: Orales System.*

Im Modulblock B werden naturwissenschaftliche und medizinische Grundlagen interdisziplinär themenorientiert parallel und eng verknüpft mit einer theoretisch praktischen Ausbildung in den zahnmedizinischen Fächern Werkstoffkunde, Prothetik, Kieferorthopädie und Zahnerhaltung angeboten. Die Studierenden werden durch das Training feinmotorischer Basisfertigkeiten und durch inhaltlich angepasste frühe klinische Simulationen an die zukünftige klinische Tätigkeit herangeführt. Im praktischen Kurs der Zahnerhaltung erlernen die Studierenden Grundlagen der zahnerhaltenden Diagnostik und der Zahnpräparation. Die Vermittlung von Basiskenntnissen über soziale Faktoren in der Zahnmedizin, Grundprinzipien der Kommunikation und erste Schritte zum wissenschaftlichen Denken und Arbeiten flankieren den Modulblock B.

#### Modulblock *C: Infektionen, Entzündungen und Prävention I, II und III.*

Im Modulblock C (C1 und C2 im 2. Abschnitt und C3 im 3. Abschnitt der Lernspirale) werden die „typischen“ zahnmedizinischen Erkrankungen Karies und Parodontopathien ebenso wie Kiefergelenkentzündungen und andere Pathologien bezüglich ihrer Ursachen, der Erkrankung selbst und deren Therapie hinsichtlich der Pathophysiologie, Pathobiochemie, Anatomie, Physiologie, Pharmakologie und Mikrobiologie vermittelt. Modul C3 fokussiert parallel zur synoptischen Behandlung I die klinische Tätigkeit an Patient:innen. In der zahnerhaltenden Ausbildung sieht das Modulkonzept die praktischen Übungen zur Prävention oraler Erkrankungen vor. Die Studierenden erlernen in der Theorie und praktisch in der Behandlungssituation den adäquaten individuellen Umgang mit Hilfsmitteln der zahnärztlichen Prophylaxe, erstellen Präventionskonzepte und instruieren sich gegenseitig in der Mundhygiene. Darüber hinaus finden entsprechend dem Schwerpunkt dieses Modulblocks praktische Übungen zur Endodontie, Parodontologie und Kariologie am Phantomkopf statt. Dieser praktische Anteil ermöglicht den Studierenden, ihre erlernten theoretischen Kenntnisse unmittelbar am Modell/Phantomkopf umzusetzen.

#### Modulblock *D: Angeborene und erworbene Zahn- und Kieferdefekte, initialer Zahnverlust I, II und III.*

Im Modulblock D, ebenfalls mit 2 Modulen im 2. Studienabschnitt und dem 3. Modul im dritten Abschnitt, stehen die klinische Untersuchung, Behandlungsplanung und Indikationen im Rahmen des periprothetischen Therapiekonzeptes im Vordergrund. Auch die Grundlagen der kieferorthopädischen Diagnostik und des Mund-Kiefer-Gesichts-chirurgischen Themenspektrums gehören zum Lehrportfolio. Modul D3 flankiert wiederum den Start der synoptischen Behandlung in F2P.

#### Modulblock *E: Zahn- und Kieferfehlbildungen I, II und III sowie Syndrome.*

Modulblock E besteht im Wesentlichen aus der Vermittlung und dem Erlernen von Zahn- und Kieferfehlstellungen sowie aus der kieferorthopädischen und Mund-Kiefer-Gesichts-chirurgischen Diagnostik und Therapie. Er startet mit E1 am Ende des ersten Teils des Abschnitts „Vom Symptom zur Erkrankung“ und wird erst im dritten Abschnitt „Therapie“ mit E2 und E3 fortgeführt, also vertieft.

#### Modulblock *F: Fortgeschrittener Zahnverlust und Zahnlosigkeit I und II.*

Modulblock F verbindet in 2 Modulen im 5. und im 8. Semester Diagnostik und das Erlernen aller Zusammenhänge zum *fortgeschrittenen Zahnverlust und der Zahnlosigkeit *(I) mit dem Erlernen und der Anwendung der *Therapie *(II) zu diesem Thema. In Modul F1 werden im präklinischen Unterricht die praktischen Fertigkeiten geschult, die für die Anfertigung von Zahnersatz bei zahnlosen und teilbezahnten Patient:innen erforderlich sind. Diese erlernten Fertigkeiten werden im klinischen Studienabschnitt in Modul F2 bei der Behandlung eigener Patient:innen angewandt und erprobt.

#### Modulblock *G: Synoptische Behandlungsplanung I: einfache Fälle und II: komplexe Fälle.*

Im Modulblock G steht das Erlernen der zahnmedizinischen Behandlungsplanung im synoptischen Behandlungskonzept im Vordergrund. Bestehend aus Modul G1, dem letzten Modul im Modulblock „Vom Symptom zur Behandlung“ und im zeitlichen Ablauf kurz vor Eintritt in den klinischen Studienabschnitt „Therapie“, werden in der Theorie echte Patientenfälle aufgearbeitet und intensiv Therapien und Behandlungsabläufe geplant. Hier finden auch – zur Vorbereitung auf den klinischen Studienabschnitt – die ersten Kontakte mit echten Patient:innen im Rahmen von Befundaufnahmen statt.

Im letzten Modul des gesamten Curriculums, Modul G2, werden komplexe Behandlungsplanungen für die von den Studierenden behandelten Patient:innen durchgeführt und somit die in den vorhergehenden Modulen erlernten Inhalte angewandt. In Vorlesungen werden weitergehende komplexe Behandlungsplanungen, die über das Spektrum der Behandlung durch Studierende hinausgehen, erlernt. Ergänzt wird die Patientenbehandlung durch allgemeinmedizinische Aspekte und Themen der Patientenbetreuung. Fächer wie Pharmakologie, Unfallchirurgie, allgemeine Chirurgie, Humangenetik und weitere sind an der Vermittlung der theoretischen und praktischen allgemeinmedizinischen Grundkenntnisse für die Examensreife beteiligt.

#### Modulblock *H: Spezifische Patientengruppen I und II: Kinder, Senioren und Beeinträchtigte etc.*

Der Unterricht im Modulblock H adaptiert die erlernten allgemeinen Inhalte der zahnmedizinischen Therapie auf die Behandlung spezifischer Patientengruppen (z. B. Kinder, Senioren, Beeinträchtigte) mit ihren jeweiligen Bedürfnissen. Der Unterricht in Modul H1 findet im 9. Semester statt. Die Inhalte werden in Modul H2 im 10. Semester vertieft.

#### Modulblock *S: Studienarbeit.*

Der longitudinale Strang Wissenschaft, der zunächst allgemein und grundlegend und anschließend sehr themenzentriert auf wissenschaftliches Arbeiten vorbereitet, bildet eine wesentliche Säule der wissenschaftlichen Ausbildung im Modellstudiengang. Eine für alle Studierenden verpflichtende wissenschaftliche Arbeit, die Studienarbeit, bildet das zweite wesentliche Element. Sie wird im zweiten Teil des 5. Semesters im gleichnamigen Modul S („Studienarbeit“) angefertigt. Das Spektrum der inhaltlichen Ausgestaltung der Studienarbeit ist bewusst breit gehalten, um den Studierenden die Möglichkeit zu geben, eine ihren Neigungen entsprechende Thematik zu bearbeiten, und reicht von der literaturbasierten Aufarbeitung des Forschungsstandes zu einem spezifischen Thema über klinische Fallberichte bis hin zur Zusammenfassung und kritischen (reviewartigen) Diskussion gesundheitspolitischer Themen.

#### Zusammenhänge zwischen Modulen.

Für die Modulblöcke G und H (ab Semester 6), die über jeweils 2 Module verfügen, sind alle davor absolvierten Module unabdingbare Voraussetzung, da in beiden Modulblöcken die zahnmedizinische und medizinische Fachvielfalt im synoptischen Sinne vermittelt, erlernt und angewandt werden soll.

Modul F2 stellt ebenso wie Modul G2 in besonderer Weise die Verzahnung von Theorie (F2T und G2T) und klinischer Anwendung (F2P und G2P) dar: Beide bilden zum Abschluss des jeweiligen Studienjahres 4 und 5 ein auch prüfungstechnisch relevantes, kombiniertes Modul mit dem Theorieanteil (F2T und G2T) sowie der longitudinal über die beiden Studienjahre verlaufenden klinischen Behandlung der Patient:innen durch die Studierenden.

Die Verknüpfung von Theorie und Praxis im jeweiligen Behandlungsjahr (F2P und G2P) gilt inhaltlich auch für die Module D3, C3 und E2 sowie H1, E3 und H2.

### Longitudinale inhaltliche Stränge

Das dritte Strukturmerkmal von iMED DENT sind longitudinale inhaltliche Stränge zum Erlernen von (i) zahnklinisch-praktischen Fertigkeiten, (ii) Wissenschaftskompetenz sowie (iii) Kommunikationskompetenz (Abb. 3).

Die Vermittlung, das Erlernen, die Anwendung sowie das Üben der zahnklinisch-praktischen Basisfertigkeiten ab dem 1. Semester sowie der präklinisch praktischen Fertigkeiten ab dem 3. Semester werden erstmals im ersten Teil des 5. Semesters im Modul F1 im Rahmen der Assistenztätigkeit während der Behandlung durch fortgeschrittene Kommiliton:innen umgesetzt. Im 6. Semester folgen die klinische und radiologische Befunderhebung sowie weiteres Training an Simulationseinheiten als Vorbereitung auf die synoptische Zahnmedizin I und II.

Ebenso longitudinal und integriert wird die zahnärztliche Kommunikationskompetenz ab Modul B2 im 2. Semester modulbezogen sowie fächerübergreifend gelehrt und mithilfe der Lernspirale durch kontinuierliches Üben und Erweiterung der Kompetenzen umgesetzt.

Der Wissenschaftsstrang beginnt bereits im 2. Semester im inhaltlichen Kontext von Modul A und setzt sich kontinuierlich fort bis zur Umsetzung des Erlernten in der Studienarbeit (Modul S) im zweiten Teil des 5. Semesters. Auch nach der Absolvierung des Moduls S wird bis zum Abschluss des Studiums die wissenschaftliche Ausbildung fortgeführt.

## Auswahl qualitätssichernder Maßnahmen

Der Einsatz qualitätssichernder Maßnahmen bildet eine der wichtigsten Grundlagen für die Optimierung des Modellstudiengangs iMED DENT.

Zu diesen Maßnahmen zählen neben anderen:

- systematische Evaluation der Lehrveranstaltungen durch Studierende und Lehrende,
- Maßnahmen zur Qualifizierung von Dozent:innen und Tutor:innen,
- kontinuierliches Feedback für Studierende bzgl. Arzt-Patienten-Kommunikation sowie zur Behandlungsqualität bei den Patient:innen,
- regelmäßige Supervision für die Studierenden zur Reflexion der eigenen Rolle und des Umgangs mit Patient:innen,
- Erstellung eines für Studierende und Lehrende transparenten Leistungsprofils und
- engmaschiges, im klinischen Studienteil tägliches, kompetenzbasiertes Feedback für die Studierenden.

Operativ werden diese Maßnahmen durch das Curriculum-Komitee (CK) iMED DENT koordiniert, dem die Leitungen aller Module sowie weitere Fach‑, Studierendenvertreter:innen und Mitarbeiter:innen des Prodekanats für Lehre angehören. Die systematische Evaluation der Lehrveranstaltungen durch Studierende erfolgt an jedem Modulende. Die Ergebnisse werden dann im Plenum des CK iMED DENT präsentiert. Kritische Punkte hinsichtlich Struktur oder Inhalt werden möglichst direkt in Arbeitsaufträge für die Modulleitungen und/oder Fächer umgesetzt oder durch Ad-hoc-Arbeitsgruppen bearbeitet.

Die weiteren Feedbackschleifen für Studierende, z. B. zur Arzt-Patienten-Kommunikation, zur Behandlungsqualität bei den Patient:innen, die regelmäßige Supervision für die Studierenden zur Reflexion der eigenen Rolle und des Umgangs mit Patient:innen sowie die Erstellung eines für Studierende und Lehrende transparenten Leistungsprofils wird durch die Lehrenden im Studienabschnitt 3 „Therapie“ studienbegleitend durchgeführt.

## Erreichung von Studiengangzielen – Optimierungsbedarf im laufenden Curriculum

Seit dem Start der ersten Kohorte im Oktober 2019 wird der Modellstudiengang aufgrund der studentischen Evaluationsergebnisse optimiert. Dadurch befinden sich sowohl die Struktur als auch die Inhalte in einem kontinuierlichen Abstimmungs- und Optimierungsprozess, der, gemeinsam mit der Umsetzung der derzeitig gültigen zahnärztlichen Approbationsordnung, bereits zu verschiedenen Änderungen der Studien- und der Prüfungsordnung geführt hat.

Im Bereich Integration, Interdisziplinarität und Interprofessionalität gibt es in mehreren Modulen weiteren Abstimmungsbedarf innerhalb der zahnmedizinischen Fächer. Vor allem zwischen den Fächern der klinischen Medizin und den zahnmedizinischen Fächern sind umfangreiche Absprachen zu Lehrinhalten, aber auch zur Integration im Stundenplan getroffen worden. Bereits gelungen ist die Integration der eigentlich bei den Studierenden eher „ungeliebten“ naturwissenschaftlichen Grundlagenfächer durch engen Bezug der jeweiligen Fachinhalte zu den parallel unterrichteten zahnmedizinischen Grundlagen. Eine deutlich höhere Zufriedenheit mit den naturwissenschaftlichen Grundlagenfächern im Vergleich zum Regelstudiengang ist also bereits zu verzeichnen. Auch die Abstimmung innerhalb der zahnmedizinischen Fächer im Modul „Angeborene und erworbene Zahn- und Kieferdefekte, Initialer Zahnverlust II“ ist bisher ausgesprochen positiv ausgefallen.

Die Optimierung der inhaltlichen und organisatorischen Integration der Einzelfächer stellt sich auch in iMED DENT als wesentliche Aufgabe für die Qualitätssicherung dar; identische Erfahrungen wurden bereits im Modellstudiengang Medizin iMED in Hamburg gemacht und waren daher für die beteiligten Entwickler:innen von iMED DENT wenig überraschend.

Die Erreichung des Studiengangziels „Früher Patient:innenkontakt“ wurde durch Kliniktage und die Betreuung in den Kursen in mehreren Modulen sehr positiv bewertet (Module B2, B3, F1, G1). Optimierungsbedarf wird hier derzeit nicht gesehen.

Auch im Bereich des longitudinalen Wissenschaftsstrangs sind sehr positive Evaluationen zu verzeichnen. Optimierungsbedarf wird daher auch für dieses Studiengangziel derzeit nicht gesehen.

Im longitudinalen Strang „Kommunikation“ erfolgt die Vermittlung der zentralen Basisfertigkeiten für die Umsetzung einer professionellen, patientenorientierten zahnärztlichen Versorgung einschließlich psychosozialer und kommunikativer Kompetenzen. Die Studierenden waren mit den Kursen des longitudinalen Kommunikationsstrangs bisher insgesamt ausgesprochen zufrieden, sodass auch für dieses Studiengangziel wenig Optimierungsbedarf besteht.

## Fazit

Im ersten integrierten Modellstudiengang Zahnmedizin iMED DENT in Deutschland studiert es sich gut – so lassen sich die häufig positiven Eindrücke der ersten Studierendenkohorten zusammenfassen. Allerdings trifft das (noch) nicht auf jedes Modul bzw. jede Veranstaltung zu. Wie bereits im Hamburger integrierten Modellstudiengang Medizin iMED beobachtet [9, 10], sind mehrere Durchgänge jedes Moduls notwendig, um die am „grünen“ Tisch geplanten Unterrichtsverläufe optimal an die Bedürfnisse der Studierenden und Lehrenden, aber auch an die Fachanforderungen anzupassen. Ein engagiertes Qualitätsmanagement, das für iMED DENT durch das CK iMED DENT verkörpert wird, spielt dabei eine herausragende Rolle. So wurden bereits in den ersten Jahren seit Start des Studiengangs wichtige Änderungen in den Modulen realisiert.
